# Barriers to Radiation-Induced *In Situ* Tumor Vaccination

**DOI:** 10.3389/fimmu.2017.00229

**Published:** 2017-03-13

**Authors:** Erik Wennerberg, Claire Lhuillier, Claire Vanpouille-Box, Karsten A. Pilones, Elena García-Martínez, Nils-Petter Rudqvist, Silvia C. Formenti, Sandra Demaria

**Affiliations:** ^1^Department of Radiation Oncology, Weill Cornell Medicine, New York, NY, USA; ^2^Department of Hematology and Medical Oncology, University Hospital Morales Meseguer, Murcia, Spain

**Keywords:** abscopal effect, adenosine, hypoxia, immunotherapy, macrophages, radiation therapy, transforming growth factor-β, tumor microenvironment

## Abstract

The immunostimulatory properties of radiation therapy (RT) have recently generated widespread interest due to preclinical and clinical evidence that tumor-localized RT can sometimes induce antitumor immune responses mediating regression of non-irradiated metastases (abscopal effect). The ability of RT to activate antitumor T cells explains the synergy of RT with immune checkpoint inhibitors, which has been well documented in mouse tumor models and is supported by observations of more frequent abscopal responses in patients refractory to immunotherapy who receive RT during immunotherapy. However, abscopal responses following RT remain relatively rare in the clinic, and antitumor immune responses are not effectively induced by RT against poorly immunogenic mouse tumors. This suggests that in order to improve the pro-immunogenic effects of RT, it is necessary to identify and overcome the barriers that pre-exist and/or are induced by RT in the tumor microenvironment. On the one hand, RT induces an immunogenic death of cancer cells associated with release of powerful danger signals that are essential to recruit and activate dendritic cells (DCs) and initiate antitumor immune responses. On the other hand, RT can promote the generation of immunosuppressive mediators that hinder DCs activation and impair the function of effector T cells. In this review, we discuss current evidence that several inhibitory pathways are induced and modulated in irradiated tumors. In particular, we will focus on factors that regulate and limit radiation-induced immunogenicity and emphasize current research on actionable targets that could increase the effectiveness of radiation-induced *in situ* tumor vaccination.

## Introduction

Immune checkpoint blockade with antibodies targeting cytotoxic T lymphocyte-associated protein 4 (CTLA-4) and programmed cell death protein-1 (PD-1) has shown durable responses in a significant portion of patients with metastatic cancer. However, patients that lack pre-existing antitumor immunity are generally unresponsive to these therapies (1). In these patients, treatment with immune checkpoint inhibitors needs to be combined with a strategy to induce *de novo* tumor-specific T cells. Recent findings have shed light on the potential of radiation therapy (RT) to induce such responses (2).

**Figure 1:**
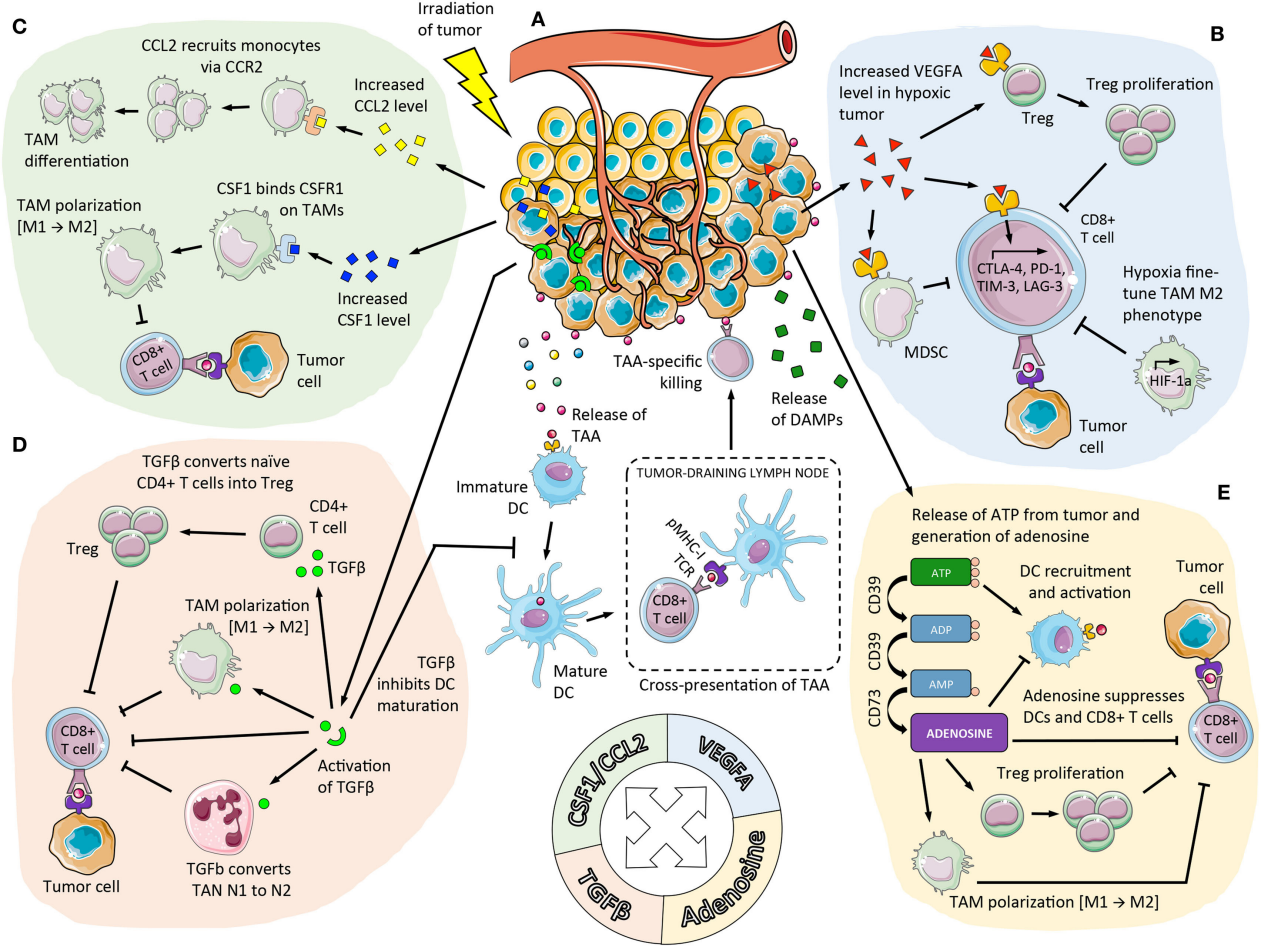
**Immunosuppressive pathways enhanced by RT in the TME that limit RT-induced *in situ* vaccination**. **(A)** DCs are recruited to the tumor and activated following RT-mediated induction of ICD and subsequent release of DAMPs in the TME [including ATP, depicted in **(E)**]. After uptake of TAAs that are released from dying tumor cells DCs become activated and migrate to tumor-draining lymph nodes where they cross-present the antigens to naïve T cells. The activated TAA-specific CD8^+^ T cells proliferate, acquire effector function, and infiltrate the irradiated tumor and abscopal sites where they eliminate tumor cells. However, RT promotes not only immune stimulation but also contributes to a suppressive TME that counteracts the newly initiated immune response. **(B)** Hypoxic regions within tumors have reduced sensitivity to RT and a suppressive TME that can be exacerbated following RT. RT upregulates transcription of HIF-1α resulting in expression of a series of genes that promote immunosuppression, by inducing Treg proliferation, M2 polarization of TAMs, and MDSC activation. **(C)** C–C chemokine receptor type 2 (CCR2)-expressing monocytes are recruited to the tumor due to increased CCL2 levels following RT. In the tumor, monocytes then differentiate to TAMs. RT can also directly modulate TAMs through induction of CSF1 causing mobilization, proliferation, and polarization of TAMs to an M2 phenotype. **(D)** RT activates latent TGFβ within the tumor that causes conversion of CD4^+^ T cells to Tregs, and polarization of TAMs and TANs to an M2 and N2 phenotype, respectively. **(E)** Tumor cells undergoing radiation-induced ICD release ATP, which is rapidly catabolized into adenosine in the TME by ectoenzymes CD39 and CD73 expressed on tumor cells, stromal cells, and immune cells. Local accumulation of extracellular adenosine suppresses DCs and effector T cells while promoting proliferation of Tregs and a more suppressive phenotype in TAMs. DC, dendritic cell; ICD, immunogenic cell death; RT, radiation therapy; DAMPs, danger-associated molecular patterns; TAA, tumor-associated antigens; TME, tumor microenvironment; pMHC-1, peptide-loaded major histocompatibility class I complex; TCR, T cell receptor; HIF-1α, hypoxia-inducible factor-1α; VEGFA, vascular endothelial growth factor A; CTLA-4, cytotoxic T lymphocyte-associated protein 4; PD-1, programmed cell death protein-1; TIM-3, T-cell immunoglobulin and mucin-domain containing-3; LAG-3, lymphocyte-activation gene 3; Treg, regulatory T cell; TGFβ, transforming growth factor β; TAM, tumor-associated macrophage; MDSC, myeloid-derived suppressor cell; CSF1, colony-stimulating factor 1; TAN, tumor-associated neutrophil; ATP, adenosine triphosphate.

Exposure of tumor cells to ionizing radiation (or certain cytotoxic chemotherapy agents) can result in immunogenic cell death (ICD) whereby upregulation or release of danger-associated molecular patterns (DAMPs) including calreticulin, high-mobility group protein B1, and adenosine triphosphate (ATP) alerts the immune system of a potential threat (3, 4). The release of DAMPs associated with RT-induced cancer cell death occurs in a dose-dependent fashion and has been shown to both recruit and activate dendritic cells (DCs) to uptake tumor antigens and cross-present them to naïve T cells thus initiating antitumor immune responses (Figure 1) (5–9). RT can also facilitate the recruitment of effector T-cells to the tumor by inducing the secretion of CXC motif chemokine ligand (CXCL)9, CXCL10, and CXCL16 by tumor cells (10–12). In addition, RT-induced upregulation of major histocompatibility complex class I molecules, FAS/CD95, and stress-induced natural killer group 2D-ligands on tumor cells enhance recognition and killing of cancer cells by cytotoxic T cells (CTLs) (10, 13–15). Overall, these RT-induced signals have been shown to mediate, at least in part, the powerful synergy between RT and a variety of immune therapeutic agents, including immune checkpoint inhibitors and DC growth factors, in experimental settings where these treatments by themselves were ineffective. The most important result of this synergy is immune-mediated tumor regression in non-irradiated metastases, known as abscopal effect, which has been seen in preclinical models as well as patients and supports the interpretation that the irradiated tumor acts as an *in situ* vaccine generating a systemic antitumor response (16–21). However, abscopal effects remain rare, highlighting the need to better understand and address the obstacles to effective *in situ* vaccination by RT.

Once tumors are established, they have evolved multiple ways to escape immune-mediated control and elimination, often by creating an increasingly immunosuppressive microenvironment (22). Myeloid cells in the tumor microenvironment (TME) are polarized toward an immunosuppressive phenotype, and DCs acquire a tolerogenic function or are excluded altogether from the tumor (23). If effector T cells are present, they are unable to function due to inhibitory molecules expressed on tumor and stromal cells and/or a suppressive cytokine milieu (22). There are multitudes of signaling pathways that govern the suppressive nature of the TME, and the modulation of these pathways by RT is an active area of study.

Tumors, which often behave like non-healing wounds, are rich in tumor-associated macrophages (TAMs), whose suppressive properties are largely regulated by colony-stimulating factor 1 (CSF1), a growth factor that is upregulated in irradiated tumors (24). TAMs secrete transforming growth factor-β (TGFβ) and other cytokines that suppress effector T cells and stimulate regulatory T cells (Tregs). The TME contains large amounts of inactive TGFβ, which can be converted to its active form by RT, as discussed below. In addition to its stimulatory effect on tumor angiogenesis, fibrosis, and cell growth, TGFβ has direct inhibitory effects on the antitumor immune response. Under conditions of hypoxic stress, which occurs commonly in growing tumors and can be further exacerbated following RT, tumor cells utilize hypoxia-inducible factors (HIFs) to induce expression of genes that help them cope metabolically with the low oxygen levels and vascularize the tumor tissue, including vascular endothelial growth factor A (VEGF-A). Moreover, the hypoxic TME contains high levels of adenosine, a pleiotropic immunosuppressive mediator that can be actively secreted from intracellular stores or generated by extracellular catabolism of ATP released following cellular stress including RT-induced ICD (5, 25). In this review, we will discuss how RT regulates these fundamental immunosuppressive pathways, how they interact and affect each other and importantly, how they modulate the ability of RT to induce antitumor immunity.

## Regulation of TAMs in the Irradiated Tumor

TAMs comprise a major component of the inflammatory infiltrate in many solid tumors and for the most part promote a tolerogenic and immunosuppressive milieu. Their presence in ovarian, prostate, cervical, and breast malignancies is correlated with poor prognosis (26). TAMs can acquire functional properties that span the spectrum from M1 to M2-type tissue macrophages. Classically activated (M1) macrophages are highly phagocytic toward tumor cells, present antigens effectively and secrete pro-inflammatory cytokines essential for the recruitment and activation of T and natural killer (NK) cells (27). In contrast, under the influence of a Th2-type cytokine environment, macrophages become alternatively activated (M2) and perform tissue remodeling and immunosuppressive functions promoting tumor progression. In most tumor studies, TAMs have been shown to promote tumor invasion and metastasis (28, 29). This pro-tumorigenic phenotype is highly influenced by the progressively growing tumor and by soluble factors secreted by both cancer cells and other infiltrating immune cells (30).

TAMs produce high levels of immunosuppressive IL-10 and stimulate angiogenesis that further supports tumor growth (31). However, in some malignancies such as lung and gastric cancer, the presence of TAMs correlated with a more favorable patient outcome, suggesting a high functional plasticity of TAMs, which may acquire M1-like properties in some tumors. Importantly, radiation can profoundly modulate TAM populations in several ways (a) it depletes TAM as well as immature myeloid cells, (b) it increases their recruitment, (c) it causes their re-distribution between areas of necrosis and hypoxia elicited by RT, (d) it changes their polarization toward either M1 or M2 phenotype, and (e) it improves the ability of macrophages to present tumor antigens (32, 33).

Although the molecular mechanisms that underlie the ability of radiation to provoke these effects remain incompletely defined, the activation of the signaling pathway mediated by the growth factor CSF1 plays a critical role. Binding of CSF1 to its cognate receptor tyrosine kinase colony-stimulating factor 1 receptor (CSF1R) rapidly initiates the proliferation, differentiation, and migration of tissue-resident macrophages (Figure 1) (34, 35). The CSF1/CSF1R pathway is critical in recruiting TAMs and promoting tumor growth. In patients with breast, prostate, and ovarian cancer, high CSF1 levels have been shown to correlate with poor prognosis (36–38). Furthermore, the prognostic value of a CSF1-responsive gene signature was validated in a subset of breast cancer patients, where it was shown to predict risk of recurrence and invasiveness (39, 40). The expression of CSF1 in a broad array of human and murine tumor cell lines was increased after irradiation *in vitro* as well as *in vivo* in implanted tumors (24). An increase in the levels of serum CSF1 was observed in prostate cancer patients receiving radiotherapy, suggesting that the radiation-induced CSF1 upregulation is clinically relevant. The molecular mechanism of RT-induced CSF1 upregulation was recently described in a mouse prostate carcinoma. The non-receptor tyrosine kinase ABL1, which mediates apoptosis and cell cycle arrest and is activated following radiation, was shown to translocate to the nucleus and bind to the CSF1 promoter region. Importantly, blocking the CSF1/CSF1R signaling pathway using either a selective inhibitor (GW2580) or a highly potent small molecule inhibitor of CSF1R kinase (PLX3397) resulted in significant reduction in TAM infiltration and improved tumor control by RT in a mouse model (24), suggesting that the CSF1/CSFR1 axis is an important therapeutic target.

Another chemokine implicated in the RT-induced myeloid cell recruitment to the tumor is C-C motif ligand 2 (CCL2). In a mouse tumor model of pancreatic adenocarcinoma (PDAC), local delivery of a single 20 Gy dose markedly augmented the release of CCL2 by tumor cells, which was consequently accompanied by the infiltration of inflammatory macrophages expressing C-C chemokine receptor type 2 (CCR2, the cognate receptor for CCL2) (Figure 1) (41). The mobilization of inflammatory monocytes *via* CCL2/CCR2 axis has been described as a negative prognosticator in breast, pancreatic, and hepatocellular cancer, and its activation may further play a key role in mediating resistance of PDAC to ablative radiotherapy (28, 42, 43). These findings suggest that CCL2/CCR2 antagonists currently under clinical evaluation may have a new role in the context of radiotherapy, where they could be used to improve patient responses (Table 1) (44–46).

**Table 1:** **Comprehensive summary of clinical trials associated with immunosuppressive pathways regulated by radiation therapy (RT)**.

Pathway targeted	Immunotherapy	RT regimen	Condition	Status and phase	Identifier
TGFβ-mediated inhibition	Galunisertib (LY2157299)—TGFβ antagonist	Stereotactic body radiotherapy	Hepatocellular carcinoma	Not yet recruiting (Phase 1)	NCT02906397

	Galunisertib (LY2157299)—TGFβ antagonist	7.5 Gy × 3 fractions	Breast cancer	Recruiting (Phase 2)	NCT02538471

	Fresolimumab (GC1008)—TGFβ antagonist	7.5 Gy × 3 fractions	Breast cancer	Ongoing (Phase 2)	NCT01401062

	Galunisertib (LY2157299)—TGFβ antagonist	1.8–2.0 Gy × 30 fractions	Malignant glioma	Ongoing (Phase 1–2)	NCT01220271

	Fresolimumab (GC1008)—TGFβ antagonist	Stereotactic ablative radiotherapy	Non-small cell lung carcinoma	Recruiting (Phase 1–2)	NCT02581787

Tumor-associated macrophages-recruitment and polarization	Pexidartinib (PLX3397)—CSF1R inhibitor	Yes (dose not determined)	Prostate cancer	Recruiting (Phase 1)	NCT02472275

	Pexidartinib (PLX3397)—CSF1R inhibitor	60 Gy (5 days/week for 6 weeks)	Glioblastoma	Ongoing (Phase 1–2)	NCT01790503

	Pexidartinib (PLX3397)—CSF1R inhibitor	No RT	Tenosynovial giant cell tumor	Ongoing (Phase 3)	NCT02371369

	Carlumab (CNTO 888)—anti-CCL2 monoclonal antibody	No RT	Prostate cancer	Completed (Phase 2)	NCT00992186

Adenosine-mediated inhibition	MEDI9447—CD73 inhibitor	No RT	Advanced solid tumors	Recruiting (Phase 1)	NCT02503774

	Tozadenant (SYN115)—A2AR antagonist	No RT	Parkinson’s disease	Completed (Phase 2–3)	NCT01283594

VEGF-A/HIF-1α-mediated inhibition	Bevacizumab—anti-VEGF monoclonal antibody	Yes (dose not determined)	Glioblastoma multiforme	Ongoing (Phase 0)	NCT01091792

	Sorafenib—protein kinase inhibitor targeting VEGF receptor	1.8 Gy daily for 5 weeks	Pancreatic cancer	Completed (Phase 1)	NCT00375310

	Bevacizumab—anti-VEGF monoclonal antibody, Temozolomid	60 Gy (5 days/week for 6 weeks)	Glioblastoma	Ongoing (Phase 3)	NCT00884741

	Bevacizumab—anti-VEGF monoclonal antibody, Ipilimumab—anti-CTLA-4 monoclonal antibody	No RT	Metastatic melanoma	Ongoing (Phase 1)	NCT00790010

## Hypoxia in RT-Treated Tumors and Immune Regulation by HIF-1α and VEGF-A

Perturbation in oxygen homeostasis is a common feature of solid tumors, in which hypoxic regions are more resistant to RT. Indeed, ionizing radiation creates free radicals that are highly reactive due to their unpaired electrons and can therefore react with molecular oxygen leading to the production of reactive oxygen species (ROS). High concentrations of ROS, such as superoxide anion radical or hydrogen peroxide, can initiate harmful chemical reactions within the cells, including DNA damage. Thus, well-oxygenated cancer cells are more sensitive to cytocidal effects of RT than hypoxic cells.

Hypoxia-inducible factor-1α (HIF-1α) is a key transcription factor induced by hypoxia that has been reported to correlate with a poor prognosis, local tumor recurrence, and distant tumor metastases after RT (47, 48). Upregulation of HIF-1α in response to RT enhances endothelial cell radioresistance (49). Irradiation induces the stabilization of HIF-1α protein in glioma cells, thereby promoting angiogenesis and malignant progression (50). HIF-1α regulates multiple genes and signaling pathways including cancer cell survival, tumor neovascularization, and metabolism, which directly and indirectly impact antitumor immunity. Hypoxia can interfere with T cell effector function by selectively upregulating programmed death-ligand 1 (PD-L1) expression on both tumor cells and myeloid-derived suppressor cells (MDSCs) in a HIF-1α-dependent manner. Blockade of PD-L1 under hypoxia prevents T cell apoptosis and abrogates MDSC-mediated T cell suppression by modulating MDSCs cytokine production (51, 52).

Accumulating evidence indicates that hypoxia can also contribute to immune tolerance by regulating immunosuppressive cell populations. Facciabene et al. have demonstrated that hypoxic tumors promote the recruitment of Tregs *via* CCL28, which, in turn, dampen effector T cell function and promote angiogenesis (53). TAMs have been shown to inhibit T-cell proliferation under hypoxia in a HIF-1α-dependent manner in the murine MMTV-PyMT model of breast cancer. Furthermore, targeted deletion of HIF-1α in myeloid cells resulted in reduced tumor growth (54). Although tumor hypoxia does not influence the differentiation and/or polarization of TAMs, it does fine-tune the phenotype of the M2-like macrophage population (55). HIF-1α also regulates MDSCs differentiation and function in the TME (51, 56). Sceneay et al. have also reported that factors secreted by hypoxic tumors (driven by HIF-1α signaling) condition the establishment of the premetastatic niche by recruiting granulocytic MDSCs and suppressing NK cell cytotoxicity (57).

As mentioned above, one important role of HIF-1α is the stimulation of angiogenesis (58–60). In the absence of oxygen, HIF-1α binds to hypoxia-response elements, thereby activating the expression of multiple hypoxia-response genes, including VEGF-A, which is produced by a majority of tumor cells, is present in the serum of cancer patients and whose expression is increased by RT (Figure 1) (61, 62). In addition to its direct pro-angiogenic properties, VEGF-A is also a potent immunosuppressive mediator in the TME. VEGFR2, one of its two key receptors, is selectively expressed by Foxp3^high^ CD4^+^ Tregs and VEGF-A has been shown to induce Treg proliferation in a VEGFR2-dependent manner in tumor-bearing mice and metastatic colorectal cancer patients (63, 64). VEGF-A arrests the differentiation of myeloid cells, resulting in the accumulation of MDSCs (65, 66). Horikawa et al. have shown recently that the VEGF-A/VEGFR2 pathway increases intratumoral MDSCs and promotes tumor progression in a mouse ovarian cancer model. They also showed that VEGF expression correlated with MDSCs infiltration in human samples from the peritoneum of ovarian cancer patients with disseminated disease (67). Besides these effects on immunoregulatory cells, a direct inhibition of conventional T cells by VEGF-A has been reported (68). VEGF-A also enhances the expression of inhibitory receptors by CD8^+^ T cells (Tim-3, CTLA-4, PD-1, Lag-3) in a VEGFR2-NFAT-dependent manner. Treatment of CT26 tumor-bearing mice with VEGF-A antibody decreases the expression of these inhibitory receptors on CD8^+^ T cells isolated from the tumor and from hepatic metastases (69). Recently, Motz et al. have demonstrated that VEGF-A together with IL-10 and PGE2 in hypoxic regions can induce Fas ligand expression on tumor endothelial cells, leading to the apoptosis of effector CD8^+^ T cells (70).

Altogether, these data suggest that VEGF-targeted therapies could reverse immunosuppression and increase antitumor immunity. Notably, inhibiting VEGF-A pathway by neutralizing antibodies has been shown to increase the antitumor effects of ionizing radiation (71, 72). Currently, the most prominent VEGF pathway-targeting drug is bevacizumab; a recombinant humanized monoclonal antibody that binds to human VEGF-A. A combinatorial therapy targeting tumor hypoxia by using HIF-1α or VEGF-A inhibitors along with RT and immunotherapy (PD-L1 or other immune checkpoint inhibitor) may be beneficial for enhancing antitumor immunity in cancer patients.

## Dual Role of Adenosinergic Signaling in Tumors Following RT

Adenosine accumulation in the TME has been identified as a central immunosuppressive factor (73, 74). ATP is the universal carrier of chemical energy and is present in all metabolically active cells. When released into the extracellular space following ICD, ATP triggers recruitment of DCs, and other antigen-presenting cells through P2Y2 receptor-dependent chemotaxis (75). In addition, ATP constitutes an important activation signal for DCs by activating the NLRP3 inflammasome through ligation with the P2RX7 receptor (76). DCs are stimulated to produce pro-inflammatory cytokines IL-1β and IL-18, and they start to differentiate, allowing them to process engulfed tumor antigens, and migrate to the draining lymph nodes to cross-present the antigens to naïve T cells (6, 77, 78).

RT has been shown to trigger release of ATP from tumor cells in a dose-dependent manner suggesting that ATP is a key mediator of radiation-induced antitumor immunity (5). However, ATP is rapidly catabolized in the TME by the action of ectonucleotidases CD39 (ecto-nucleoside triphosphate diphosphohydrolase 1) that catalyzes the hydrolysis of ATP into adenosine diphosphate (ADP) and ADP into adenosine monophosphate (AMP). AMP is then converted into adenosine by irreversible hydrolysis catalyzed by CD73 (ecto-5′-nucleotidase), the rate-limiting enzyme for adenosine generation (79). Adenosine is a pleiotropic anti-inflammatory mediator that directly inhibits the activity of antigen-presenting cells and effector lymphocytes, primarily through uptake *via* adenosine receptor 2A (A2AR), and also indirectly by promoting proliferation of Tregs and skewing the polarization of TAMs from an M1 to an M2 phenotype (Figure 1) (80–82). Moreover, the expression of A2AR is upregulated under hypoxic conditions (83).

CD73 is expressed in a multitude of cancers and its significance in tumor progression is supported by studies showing that CD73 expression levels correlated with worse prognosis in triple-negative breast cancer as well as in gastric, colorectal, and gallbladder cancer (84–87). Moreover, preclinical studies have revealed that CD73-deficient mice have a suppressed growth of implanted tumors and are protected from experimental metastases (88). Although the expression of CD39 has not yet been correlated with tumor behavior or stage in patients, CD39 is overexpressed in some human tumor cells and co-culture of CD39^+^ tumor cells with activated CD4^+^ and CD8^+^ T cells suppressed T cell proliferation, which was abrogated in the presence of CD39-blocking antibody or A2AR inhibitor (89). Interestingly, CD39 and CD73 are also expressed by effector T cells, and their expression is regulated by the concentration of ATP metabolites in the extracellular milieu (90). Expression of CD39 and CD73 in Tregs correlates with their suppressive capacity, highlighting the plasticity and importance of adenosinergic signaling in regulating immune activation (91–94). Moreover, MDSCs express CD39 and CD73 and are sensitive to adenosine signaling, which affects their function and migration (95, 96). The suppressive activity of granulocytic MDSCs is increased in presence of AMP *in vitro* (97).

Stagg and colleagues have shown that pharmacological blockade of adenosine generation or uptake, by inhibition of CD73 or A2AR, respectively, promotes antitumor immune responses and synergizes with anti-PD-1 and anti-CTLA-4 (98–100). To date, little is known of the interplay between radiation and adenosine-mediated immunosuppression. However, our data suggest that the dose-dependent release of ATP following tumor irradiation along with a high ectonucleotidase expression in the TME may lead to increased adenosine levels following RT and limit the efficacy of radiation-induced *in situ* tumor vaccination (101).

## TGFβ as a Central Regulator of RT-Induced Tumor Immunogenicity

TGFβ is a multipotent cytokine involved in the regulation of cellular differentiation, survival, and function of many, if not all, immune-cell types (102–106). For instance, approximately 25 years ago, Shull and colleagues reported a massive activation and expansion of T cells in TGFβ1-deficient mice, indicating that one of the major roles of TGFβ is the regulation of T cell differentiation and function (107). Since then, TGFβ has been demonstrated to inhibit the functional differentiation of CD8^+^ T cells into CTLs and to actively contribute to the conversion of naïve CD4^+^ T cells into Tregs upon TCR cross-linking (108–110). TGFβ has also been reported to induce expression of CD73, and to a lesser extend CD39 in both CD4^+^ and CD8^+^ T cells (111). Chalmin and colleagues have corroborated these findings by showing that the expression of CD39 and CD73 is under TGFβ transcriptional control in *in vitro* generated Th17 cells *via* Stat3 activation (112).

Immune regulation mediated by TGFβ extends far beyond the T cell compartment with TGFβ playing a key role in subverting adaptive immunity by inhibiting DCs activation and skewing the phenotype of macrophages from M1 to M2 (113–116). Importantly, aside from their well-described role in host defenses, accumulating evidence indicate that neutrophils exhibit a high phenotypic and functional plasticity depending upon TGFβ available in the TME (117). Indeed, similar to macrophages, TGFβ has been shown to drive the phenotype change of a more tumor cytotoxic and pro-inflammatory phenotype (N1) into a tumor supportive phenotype (N2) (113). Radiation activates latent TGFβ through a conformational change of the latency-associated peptide–TGFβ complex releasing active TGFβ (Figure 1) (118, 119).

The role of TGFβ as a master regulator of RT-induced antitumor T cell responses was demonstrated in two mouse tumor models of breast cancer. Antibody-mediated neutralization of TGFβ was required to achieve RT-induced priming of CD8 T cells to multiple endogenous tumor antigens. Importantly, complete regression of the irradiated 4T1 tumor and inhibition of spontaneous lung metastases was seen only in mice treated with RT in the presence of TGFβ neutralization and was mediated by T cells. Likewise, effective growth inhibition of non-irradiated synchronous subcutaneous TSA tumors required TGFβ neutralization together with RT to the contralateral TSA tumor, demonstrating an abscopal effect (120). These data highlight the importance of TGFβ-mediated immunosuppression in the context of the irradiated tumor. While concurrent blockade of TGFβ with RT-achieved therapeutically effective antitumor immune responses able to extend mice survival, upregulation of PD-L1 in the irradiated tumor, detected on both carcinoma cells and infiltrating myeloid cells, was found to limit tumor rejection, leading to early tumor recurrence. Upregulation of PD-L1-following RT has been reported in several preclinical studies and is mediated *via* at least two distinct mechanisms. In relatively immunogenic tumors, RT alone was able to elicit antitumor T cells that infiltrated the tumor and produced interferon-γ (IFNγ), which in turn induced PD-L1 expression on tumor cells (120, 121). Similarly, PD-L1 upregulation was driven by effector T cell infiltration in a poorly immunogenic tumor after RT plus TGFβ blockade (120).

These data suggest that when RT alone or in combination with an immune modulator elicits T cell responses that are insufficient to reject the tumor, the upregulation of immune checkpoint molecules in response to immune attack limits tumor rejection (122). As discussed above, another mechanism of PD-L1 upregulation is mediated by RT-induced HIF-1α (51, 123). Thus, PD-1/PD-L1 axis may represent an important obstacle to RT-induced tumor rejection, a hypothesis currently being tested in several clinical studies (124).

## Using Radiotherapy to Enhance Responses to Immunotherapy in the Clinic

Several therapeutics designed to counteract the accumulation or action of immunosuppressive mediators are undergoing testing in cancer patients, in some cases in combination with RT. Table 1 provides examples of clinical trials that investigate drugs targeting the suppressive pathways discussed above. We have not included trials testing RT with anti-PD-1 or anti-PD-L1 since the latter were discussed in several recent reviews (124–126).

Antiangiogenic therapy in the form of the anti-VEGF-A antibody bevacizumab has been tested in combination with the anti-CTLA-4 antibody ipilimumab in patients with metastatic melanoma demonstrating favorable clinical outcomes and was associated with improved tumor T cell infiltration (127, 128). Preclinical studies in colorectal cancer xenografts have demonstrated that inhibition of the VEGF receptor (VEGFR) with concomitant fractionated RT resulted in normalization of vasculature and improved tumor control compared to RT or VEGFR-inhibition alone (129). Hyperfractionated RT is currently being combined with bevacizumab in glioblastoma patients and with sorafenib (a protein kinase inhibitor targeting VEGFR) in patients with pancreatic cancer (NCT00884741, NCT00375310).

The central role of TGFβ in modulation of RT-induced tumor immunogenicity has prompted the combined use of RT and TGFβ-inhibitors in clinical cancer trials. Following the development of the small molecule inhibitor of TGFβ-receptor I galunisertib (LY2157299), its safety profile has been tested in clinical trials, where intermittent administration was shown to be safe in patients with advanced cancer (130, 131). TGFβ neutralization by the monoclonal antibody Fresolimumab (GC1008) was also shown to be without dose-limiting toxicity up to 15 mg/kg in malignant melanoma and renal cell carcinoma (132). Fresolimumab is currently being tested in combination with hypofractionated RT in patients with metastatic breast cancer and lung cancer (NCT01401062, NCT02538471).

Inhibition of TAM recruitment or activation in solid tumors as a measure to reduce immune suppression and favor immune-mediated antitumor activity is a promising therapeutic concept (36, 133). A phase I–II study of the CSF1R inhibitor PLX3397, which included 23 patients with advanced tenosynovial giant-cell tumors in the extension phase II part, showed promising results, with 12 patients having a partial response and 7 patients with stable disease. The median duration of responses was 8 months at the time of data cutoff (134). The CSF1R inhibitor PLX3397 is currently under investigation in patients with prostate cancer and glioblastoma in combination with RT (NCT02472275, NCT01790503). Moreover, safety and tolerability of an anti-CCL2 monoclonal antibody (carlumab, CNTO 888) as single therapy is under investigation in metastatic and castrate resistant prostate cancer (NCT00992186).

Although adenosine blockade has not been clinically tested in patients receiving RT, inhibitors of both adenosine conversion (anti-CD73 monoclonal antibodies) and adenosine uptake (A2AR-inhibitors) have been tested for safety and tolerability in patients with cancer and Parkinson’s disease, respectively (NCT02503774, NCT01283594). Also in development for potential use in cancer patients are antibodies targeting CD39, which could potentially provide the advantage of increasing extracellular ATP released during RT-induced ICD while simultaneously limiting the generation of adenosine precursors (135).

## Conclusion

The use of localized RT as an adjuvant to immunotherapy with the goal of inducing *in situ* tumor vaccination is a promising concept for the treatment of cancer patients who lack a pre-existing immune response against their tumor. However, successful induction of antitumor immunity by RT is dependent upon the balance of the pre-existing immunosuppressive factors, and the immunosuppressive and immune-activating signals that are generated by RT. Improved understanding of the specific pathways that are enabled by RT, and of their mode of action, provides several novel actionable targets for inhibition to augment radiation-induced tumor immunogenicity. More studies are warranted to determine how to best leverage the new role of RT as an inducer of antitumor T cells.

## Author Contributions

EW designed and wrote the manuscript. CL, CV-B, KP, EG-M, and N-PR contributed to writing the manuscript and preparing the illustration and table. SD and SF edited the manuscript. All authors have read and approved the final version of the manuscript.

## Conflict of Interest Statement

The authors declare that the research was conducted in the absence of any commercial or financial relationships that could be construed as a potential conflict of interest.
